# Getting the Picture: Iconicity Does Not Affect Representation-Referent Confusion

**DOI:** 10.1371/journal.pone.0107910

**Published:** 2014-09-23

**Authors:** Marina C. Wimmer, Elizabeth J. Robinson, Laura Koenig, Emma Corder

**Affiliations:** 1 University of Plymouth, School of Psychology, Cognition Centre, Plymouth, United Kingdom; 2 University of Warwick, Department of Psychology, Coventry, United Kingdom; 3 Oxford Brookes University, School of Psychology, Oxford, United Kingdom; Durham University, United Kingdom

## Abstract

Three experiments examined 3- to 5-year-olds' (*N* = 428) understanding of the relationship between pictorial iconicity (photograph, colored drawing, schematic drawing) and the real world referent. Experiments 1 and 2 explored pictorial iconicity in picture-referent confusion after the picture-object relationship has been established. Pictorial iconicity had no effect on referential confusion when the referent changed after the picture had been taken/drawn (Experiment 1) and when the referent and the picture were different from the outset (Experiment 2). Experiment 3 investigated whether children are sensitive to iconicity to begin with. Children deemed photographs from a choice of varying iconicity representations as best representations for object reference. Together, findings suggest that iconicity plays a role in establishing a picture-object relation *per se* but is irrelevant once children have accepted that a picture represents an object. The latter finding may reflect domain general representational abilities.

## Introduction

Pictures are ubiquitous features in our lives. They provide both children and adults with information about objects and events with which they are unfamiliar, have not experienced directly, and perhaps never will experience. Is this the leaf of a birch or a beech tree? How do the planets lie in relation to each other? What did dinosaurs look like? When adults interpret pictures in such circumstances, they have no difficulty treating them as representations of something. The unthinking assumption of parents and teachers is probably that children share this adult conception of pictures. They may focus on whether or not a child can identify what a picture shows, without giving thought to just how the child construes the relationship between the picture and its real (or imagined) referent. Yet as summarised below, there is evidence of surprising errors, sufficient to suggest that many 3-year-olds, despite being adept at interpreting the content of pictures, do not yet hold an adult-like understanding of pictures as representations. Evidence for representational deficits comes from studies demonstrating that 3-year-olds have difficulty to infer a hidden object's location in a room from a picture/map/model [1], [2], [3], [4], [5] and their confusions between what is shown on the picture (e.g., a doll with a star sticker) and how the referent looks like here and now (i.e., doll with a butterfly sticker) [6], [7], [8]. The aim of the current research was to examine why picture-object referential confusions emerge in 3- to 5-year-old children.

One sign of failure fully to understand the representational relationship between pictures/models and their referents is 2- and 3-year-olds' poor handling of geometric correspondences between objects in real spaces and their picture/model representations in order to infer a hiding location [1], [2], [3], [4], [5]. However, in these tasks the relationship between a picture and (e.g.) a hiding location is arbitrary; children do not have to understand why the two spaces correspond. That is, at the beginning of these experiments children are usually informed and given ample opportunity to learn that objects in the picture are in the corresponding location in the other (real) space. In contrast, the relationship between a picture and what it represents in real life is not arbitrary. Moreover, theoretically, an understanding of correspondence does not require children to understand that one represents another [9], [10]. In particular, matching is unlike representing (e.g., in neighbouring terraced houses matching room arrangements do not imply that they represent each other) [9].

More direct evidence that 3-year-olds lack adult-like understanding of pictures as representations is their tendency to assume that changes in the real referent will be accompanied by parallel changes in the representation and *vice versa* [6], [7], [8], [11], [12], [13], [14]. For example, if a drawing is made of a teddy bear, which is subsequently given a ribbon, children may assert that the original drawing now bears a ribbon too [7]. Recently, these findings have been extended and 3- and 4-year-old children match picture-actions to changes in the real-world referent. If water is poured over a photograph of a cotton pad, children are more likely to select the real-world referent corresponding to the picture state (i.e., real wet cotton pad) [6]. Thus, a false causal relationship is ascribed to picture-object relations where action to the former has direct effects on the latter [6]. The aim of this research was to examine why these picture-referent confusions occur.

Initially it has been suggested that children make picture-object confusion errors because they have difficulty understanding whether the experimenter wants an answer in terms of reality or the picture [15]. However, referential confusions are only confined to situations in which a picture represents an object [14] ruling out an account in terms of a general misunderstanding of the reference of the question.

An alternative explanation is that children make these errors because they have difficulty holding in mind the picture's features as distinct from those of its real referent [7]. That is, children's referential confusions may be explained in terms of a source-monitoring error. Source-memory refers to the ability to distinguish between memories based on the origin of those events [16] and develops rapidly between 4- and 6-years [17], [18], [19]. Children might have difficulties in remembering the source of information, explaining picture-referent confusions.

In contrast, it has been suggested that children do not treat photographs as static representations *per se* but “link” them to their referents [6]. Children do not actively believe that the referent changes in accordance with its representation rather pictures “share properties of their referents more fluidly” [6]. Evidence for this account comes from the finding that children ascribe physical changes to a picture (e.g., wetting) to a real-world referent. It is not the case that children have difficulties in treating features of the representation as distinct from the real-world referent [7] but rather children incorporate changes to the representation into the real-world-referent [6]. Thus, low-level perceptual cues drive these confusion errors.

Furthermore, it has been suggested that children do not simultaneously treat the current picture state as a “thing in itself” and a representation of an object [14], [20]. That is, it is insufficient to treat the picture either as an object in itself or to understand its relationship to the real world scenario, something that is already mastered at 2 years [21]. Rather, children need to understand the representational relationship between the picture in its current state and the real world it refers to at the time the picture was taken. Understanding of the representational relationship between a symbol and what it refers to appears to be domain general [22], [23]. Between 3- and 4 years children develop an understanding that a pictorial stimulus such as Jastrow's duck/rabbit [24] can have two interpretations [23], [25]. This representational pictorial understanding is related to understanding linguistic symbols, such as synonyms, two words can refer to one meaning (i.e., something can be called both “bunny” and “rabbit”) and homonyms, one word can refer to two meanings (i.e., “bat” can be a flying mammal and a piece of sports equipment) as well as false beliefs [23], [26], [27], [28]. Similarly, understanding the relation between a current picture state and the situation it refers to may be part of general representational development. Indeed, one sign that this is a plausible hypothesis is the finding that referential confusions occur for both pictures and words misrepresenting reality [14]. For example, 3- to 4-year-olds assume that both a written name and a picture of a sticker change in accordance to changes in the real referent. However, these referential confusions are less likely to occur for words than pictures [14]. Nevertheless, this finding may indicate that representation-object referential confusions reflect domain-general representational developments.

The current research examined which of these accounts may best explain 3- to 5-year-olds' representation-referent confusions by manipulating *iconicity* of the representation (i.e., photograph versus colored drawing versus schematic drawing). Iconicity refers to the degree of picture-referent resemblance [29]. For assessing an understanding of picture-referent relationships, methodologies were used that are established in the literature as in Robinson et al. [7]. It was examined whether children's referential confusions depend on iconicity when the referent changed after the picture had been taken/drawn (Experiment 1) and when the referent and the picture were different from the outset (Experiment 2). In Experiment 3 it was investigated whether children are sensitive to iconicity *per se*. To examine representational developments, the important novelty was that different levels of iconicity (photograph, colored drawing, schematic drawing) represented the same real world referent.

The source memory account (i.e., the ability to distinguish between memories based on the origin of those events) suggests that the more distinctive and perceptually detailed different states are, the better the memory and later source decision [16]. Therefore, if children suffer from difficulty holding the representation distinct from the referent then the more iconic the representation is (i.e., degree of resemble to the real world referent), the more likely the child is to confuse representation and referent. If source-monitoring development accounted for referential confusion then it should be more likely to occur in photographs than colored drawings and in turn schematic drawings. Moreover, one would expect these errors to be related to general memory performance (memory control question Experiment 1).

Similarly, if low-level perceptual cues underlay picture-referent confusions [6] then one would expect differences in the magnitude of the confusion errors for different iconicity levels. That is, highly iconic pictures (i.e., photographs) should elicit more picture-object confusion errors than drawings as these have the highest perceptual similarity to the real-world referent and thus, share more properties of the real-world referent. The difference from the source-monitoring account is that no effects of memory would be expected. Specifically, picture-object confusions should occur, independent of the memory demands of holding the picture-object features in memory but should emerge more frequently for photographs than drawings.

Alternatively, if conceptual representational development underlay referential confusions [14], [20] then perceptual factors (i.e., iconicity) should have no effect and they should occur equally likely for photographs and types of drawings. In other words, a conceptual understanding of a current picture state and its relation to the referent should be unaffected by how well the picture depicts the real world.

## Experiment 1

The suggestion is that younger children treat picture-referent relationships as asymmetrical, where reality is stable and the picture is unstable. For example, if a picture represents a doll with a banana sticker and later the doll replaces the banana sticker with a star, younger children are more likely to say that on the picture the doll has now a star sticker too [7].

If children's referential confusions are a result of difficulty holding in mind pictorial features as distinct from the referent, then according to the source-monitoring account [16] the degree of resemblance of representation (i.e., iconicity) should affect confusion. Similarly, if low-level perceptual cues drive confusion errors then we would expect an effect of iconicity. Both accounts would predict that children should show more referential confusion when the picture is a photograph compared to a colored drawing and in turn a schematic drawing. Moreover, the source-monitoring account would predict picture-referent confusions to be linked to memory performance.

Alternatively, if conceptual representational developments underlie referential confusions then, once the picture-object relation is established, perceptual features (iconicity) should have no effect.

### Method

#### Participants

In total 205 children (101 females) took part; 74 3-year-olds (*M* = 3.6, *SD* = 4 months), 71 4-year-olds (*M* = 4.5, *SD* = 7 months) and 60 5-year-olds, (*M* = 5.4, *SD* = 3 months). Children were predominantly Caucasian and middle class. An additional 11 children were excluded due to lack of attention and experimenter error. All children were tested following written parental consent and their own oral assent on the day of testing. Ethical approval for this study was obtained from the Research Ethics Committee at Plymouth University.

#### Design

Each child received 2 conditions, each consisting of two “change doll” trials and two “change picture” trials. Each child entered one of three iconicity conditions (photograph, colored drawing, schematic drawing) at random.

#### Materials

**Figure 1:**
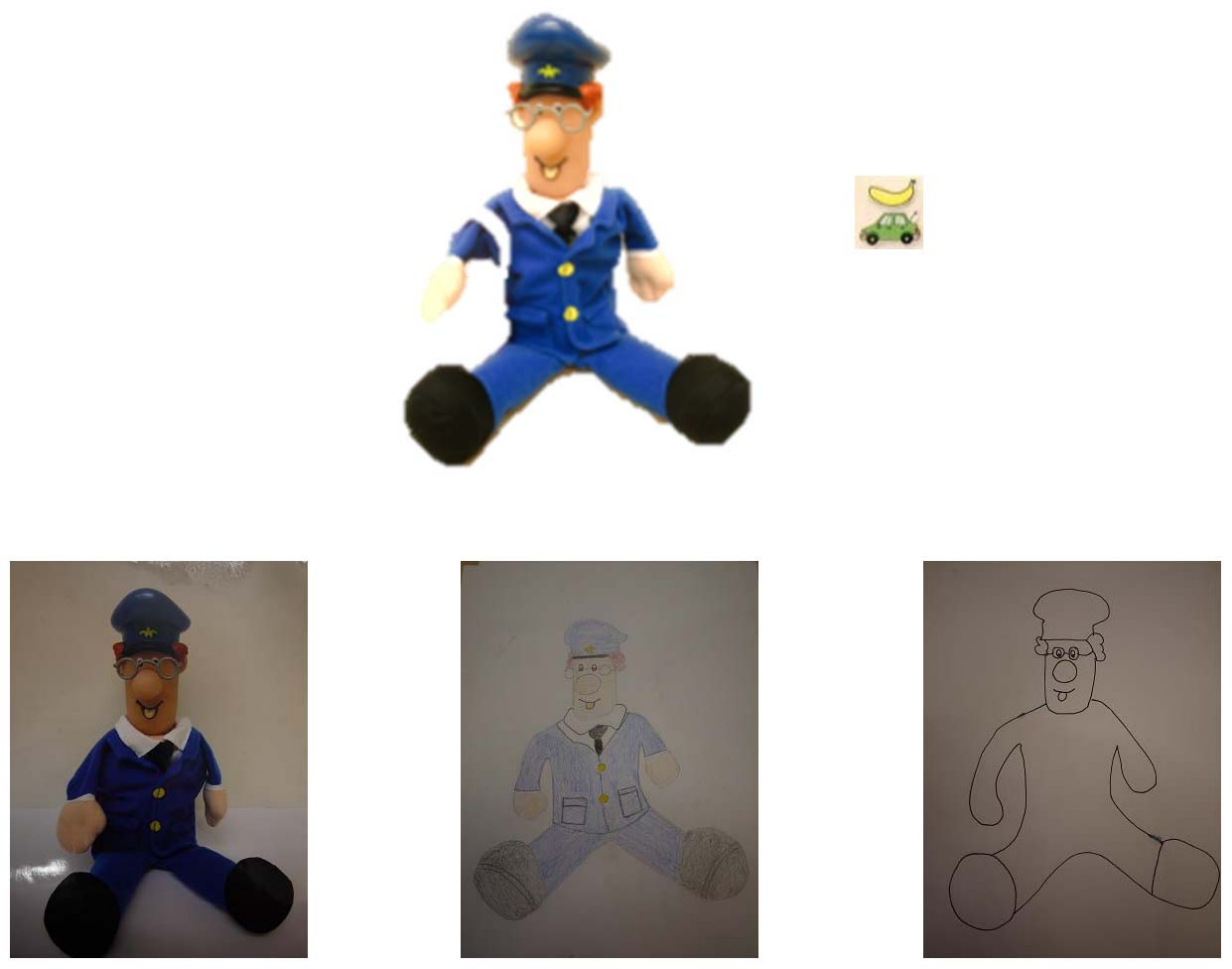
Postman Pat, stickers, and pictures of different iconicity (photograph, colored drawing, schematic drawing). Note: in Experiment 1 Postman Pat doll is shown without his bag, whereas in Experiment 2 Postman Pat doll contained his bag.

Four doll characters (Postman Pat, Jess the cat, two baby dolls called Sally and Lucy) and 12 colored stickers (butterfly, apple, bus, heart, moon, cat, car, teddy, banana, boat, dog, and flower) were used. Three stickers were used with each doll, one of them served as distractor (see Figure 1 as an example). In addition, pencils, coloured pens, and photographed stickers were used to draw/put the sticker on the picture.

#### Procedure

In the introduction phase, children were shown (e.g.) Sally the baby doll and, either a photograph of Sally, or coloured drawing or schematic drawing (depending on the iconicity condition). Then they were told, “This is a little girl called Sally, and here are different pictures (children were shown 2 pictures of similar looking characters, one being the correct one). Can you see the picture of Sally?” After the child identified the correct picture, the experimenter said, “Yes, that's right, that's a picture of Sally,” and the other picture was removed. All children identified the correct picture at this stage. Then the experimenter presented three stickers, “Look, we've got these stickers for Sally. What do you see?” After the child's response, Sally was given the boat sticker: “Sally is going to put a sticker on her dress now. Doesn't that look nice? What is it? … Yes, you are right it's a boat … Sally wants us to draw her sticker on her picture (or “Sally wants us to put a photographed sticker on the picture”). In the *schematic drawing condition* a boat sticker was drawn on the schematic drawing with a pencil, in the *colored drawing condition* a boat sticker was drawn on the colored drawing using colored pencils. In the *photograph condition* a photographed boat sticker was put on the photograph.

Then either the *changed doll* or *changed picture* trials followed.

*Changed doll.* The picture was faced down, and the experimenter said, “Now, let's give Sally a different sticker, let's give her this one instead. The boat sticker was removed and replaced by a butterfly. “Doesn't that look nice? What is it?” …Yes, it's a butterfly.” Then the doll was turned away and the test questions were asked.

*Changed item*: Remember Sally (pointing briefly), what sticker is on Sally's t-shirt?

*Unchanged item*: Remember this picture (pointing briefly), what sticker is drawn on the t-shirt?

*Memory*: Remember at the beginning, what sticker did we put on Sally's t-shirt first of all?

Children were either asked about the changed item first and the unchanged item second and *vice versa*. The memory question was always the last question. Then the second change doll trial followed, with the same instructions but different stickers and another character.

*Changed picture*. Here, the doll was faced down. The child was told, “Now let's draw something else in the picture, let's draw this instead”, (or “Now let's put a different sticker in the picture, let's take this instead.”). The boat sticker was erased/removed and a butterfly was drawn/put on its place. “Doesn't that look nice? What is it? …. Yes, that's right it's a butterfly”. Then the picture was faced down and the test questions followed.

*Changed item*: Remember this picture, what sticker is drawn on the t-shirt?

*Unchanged item*: Remember Sally, what sticker is on Sally's t-shirt?

*Memory*: Remember when we saw the picture in the beginning, what sticker did we draw/put on the t-shirt first of all?

This was continued with the second changed picture trial using different stickers and another character.

Order of test questions (changed or unchanged item question first), order of condition (change doll or change picture first) and the puppet characters used per condition were counterbalanced across participants.

### Results and Discussion

Each child received two change doll and two change picture trials and two memory questions, thus, scoring between 0 and 2 in each. Mean scores for each question per condition are displayed in table 1.

**Table 1:** Mean Scores (Range  = 0–2) on the test and memory questions per age and condition.

		Changed Item	Unchanged Item	Memory
**3-year-olds**				
Schematic (N = 23)	Change Doll	1.13	.78	.52
	Change Picture	1.00	.96	.52
Coloured (N = 24)	Change Doll	.71	.37	.42
	Change Picture	.75	.96	.42
Photo (N = 26)	Change Doll	.96	.31	.58
	Change Picture	.88	.69	.54
**4-year-olds**				
Schematic (N = 28)	Change Doll	1.50	.93	1.21
	Change Picture	.89	1.39	1.11
Coloured (N = 18)	Change Doll	1.06	.72	1.06
	Change Picture	1.00	1.06	.61
Photo (N = 25)	Change Doll	1.40	.92	.84
	Change Picture	1.04	1.36	1.12
**5-year-olds**				
Schematic (N = 23)	Change Doll	1.52	1.35	1.52
	Change Picture	1.43	1.30	1.26
Coloured (N = 17)	Change Doll	1.82	1.24	1.47
	Change Picture	1.47	1.47	1.59
Photo (N = 20)	Change Doll	1.80	1.35	1.09
	Change Picture	1.60	1.20	1.55

#### Changed Item

To examine whether it was easier to recall what sticker was on the doll or the picture after it had been changed, a 2 (changed item: doll vs. picture) ×3 (iconicity: photograph vs. colored drawing vs. schematic drawing) ×3 (age group: 3- vs. 4- vs. 5-year-olds) ANOVA on mean changed item response scores was conducted where the changed item type was the within subject variable and representation and age group were between subjects factors.

Recall of the changed item's original sticker was better when the doll was changed (*M* = 1.3) than the picture (*M* = 1.1), *F*(1, 195)  = 8.91, *p* = .003, ηp^2^ = .04. Thus, children make more errors in answer to the question about the picture than the real-world object (see also Robinson et al., 1994). Moreover, 3-year-olds (*M* = .91) performed worse than 4-year-olds (*M* = 1.15, *p* = .015) who in turn performed worse than 5-year-olds (*M* = 1.6, *p*<.001, LSD post-hoc), *F*(2, 195)  = 23.87, *p*<.001, *ηp^2^* = .20. Finally, recall of the changed item was comparable across picture iconicity (photograph: *M* = 1.28 vs. colored: *M* = 1.25 vs. schematic: *M* = 1.25), *F*(2, 195)  = 1.06, *p*>.05, *ηp^2^* = .011. This finding suggests that children are no more referentially confused the more perceptually similar or abstracted a picture is from its current real world referent.

#### Unchanged Item

To examine whether it was easier to recall what sticker was on the unchanged doll or the picture a 2 (unchanged item: doll vs. picture) ×3 (iconicity: photograph vs. colored drawing vs. schematic drawing) ×3 (age group: 3- vs. 4- vs. 5-year-olds) ANOVA on mean response scores was conducted with unchanged item type as within subject variable and the latter two as between subjects factors.

In line with Robinson et al. [7] recall of the unchanged item's original sticker was better for the doll (*M* = 1.2) than the picture (*M* = .86), *F*(1, 195)  = 15.69, *p*<.001, *ηp^2^* = .074, see also [14]. Thus, the picture-object relation is asymmetrical where reality is stable and the picture is unstable. Overall, 3-year-olds (*M* = .68) performed worse than 4-year-olds (*M* = 1.06, *p*<.001) who in turn performed worse than 5-year-olds (*M* = 1.32, *p* = .016), *F*(2, 195)  = 19.90, *p*<.001, *ηp^2^* = .17. However, both main effects were qualified by an age x unchanged item interaction, where for both 3- and 4-year-olds recalling the doll's sticker was easier than the picture's (*p* = .001, *p*<.001, respectively) but not for 5-year-olds (*p*>.05), *F*(2, 195)  = 3.34, *p* = .038, *ηp^2^* = .033. Recall was unaffected by iconicity (photograph: *M* = .97 vs. colored: *M* = .97 vs. schematic: *M* = 1.11), *F*(2, 195)  = 1.48, *p* = .231, *ηp^2^* = .015.

#### Memory

In line with Robinson et al. [7] children performed poorly on the memory question. Thus, we were unable to use it as a control for remembering the changed items' original features.

It was of further interest whether memory for the dolls' and pictures' original features was affected by pictorial iconicity and age. A 2 (item: doll vs. picture) ×3 (iconicity: photograph vs. colored drawing vs. schematic drawing) ×3 (age group: 3 vs. 4 vs. 5) ANOVA on mean memory scores revealed main effects for age group only, where 3-year-olds (*M* = .50) remembered original features more incorrectly (*p*<.001) than both 4-year-olds (*M* = .99, *p*<.001) who in turn were more incorrect than 5-year-olds (*M* = 1.5, *p*<.001), *F*(2, 195)  = 50.33, *p*<.001, *ηp^2^* = .34. There were no other main effects (all *F*s<1).

As the memory question was equally poor for both questions referring to the original picture's sticker and doll's sticker whereas performance on the changed/unchanged item was not (i.e., recall of the doll's sticker was easier than the picture's), memory failure cannot account for performance on the changed/unchanged item questions, see also [7], [30] for similar findings, posing difficulties for a source-monitoring account. Also, in line with previous findings, the picture-object relationship is asymmetrical where the picture is unstable and reality is stable [7], [14]. That is, children made more errors when recalling the picture's original sticker than the doll's. The picture-object confusions are difficult to explain within both the source-monitoring framework and low-level perceptual accounts as they would have predicted more confusion between referent and representation the higher the degree of resemblance (iconicity).

In sum, these findings suggest that iconicity of the pictorial representation has little effect on picture-object referential confusion. Once the picture-object relation is established, it is irrelevant how well the picture represents the real world referent. This finding may indicate that domain general representational developments underlie referential confusion.

## Experiment 2

If children accept that a photograph is of a particular real object they tend to over-endow the photograph with visual properties of that referent [7]. For example, if a photograph represents a teddy without a scarf and the real teddy has a scarf, children are likely to say that on the photograph the teddy has a scarf too [7].

In Experiment 1 the real referent changed after the picture was developed/drawn and children had to judge whether the changed feature appeared in the picture. In Experiment 2 the feature of the real referent was missing from the picture from the outset and the child had to recall whether that feature was in the picture. This allowed focusing on the picture as distinct from its real referent from the beginning.

Again, if source-monitoring difficulties or low-level perceptual cues underlie referential confusions then iconicity should have an effect. Alternatively, if conceptual representational developments underlie referential confusion then iconicity should have no effect.

### Method

#### Participants

In total 140 children (74 girls) took part; 69 3-year-olds (*M* = 3.7, *SD* = 3 months), 47 4-year-olds (*M* = 4.4, *SD* = 3 months), 24 5-year-olds (*M* = 5.3, *SD* = 3 months). Children were predominately Caucasian and middle-class, recruited via local schools and nurseries. They were tested following written parental consent and their own oral assent on the day of testing. Ethical approval for this study was obtained from the Research Ethics Committee at Plymouth University.

#### Design

Each child received three iconicity trials consisting of one schematic drawing, one accurate colored drawing and one photograph trial. The question order, the order of the used objects and iconicity trial order were counterbalanced across participants.

#### Materials

A cup and a spoon, Postman Pat and his bag, and Lucy the baby doll and her hat were used. Each object had three different iconicity types (schematic drawing, colored drawing and photograph). Pictures always depicted the objects without the extra items (e.g., Postman Pat without his bag) (Figure 1).

#### Procedure

First children were shown (e.g.) Lucy (doll) with her hat and a picture of Lucy (without hat) and of a similar doll. “Can you see the picture of Lucy?” After the child identified the correct picture the experimenter said, “Yes, that's right, that's a picture of Lucy!” and the similar doll picture was removed. Then the first control question was asked, “Look at the picture, has she got a mouth in the picture?” and the picture was turned down. To draw the child's attention to the object, the experimenter said, “Now let's do this” and lifted and replaced the hat. Then the second control followed: “Remember the picture; has she got two hands in the picture?” (pointing briefly at the picture). Finally, in the test question children were asked: “Remember the picture; has she got a hat in the picture?” (pointing briefly at the picture). In the end the children were asked to point to Lucy's hat.

The same procedure followed with the cup where the spoon was missing on the picture from the outset and with Postman Pat where his bag was missing from the outset.

Iconicity was manipulated within participant, thus, each child received one schematic drawing, one coloured drawing and one photograph trial. Which object appeared in which iconicity type was counterbalanced between participants.

### Results and Discussion

#### Preliminary Analysis

In total, 38 children (3-year-olds: *N* = 21; 4-year-olds: *N* = 15; 5-year-olds: *N* = 3) failed at least one of the 6 control questions. They were excluded from subsequent analyses as the source of failing the test-question (memory, lack of attention vs. picture-referent confusion) would have been difficult to determine. However, when these children were included in the analysis then the same result patterns emerged.

*Recall of features in the representation.* To examine whether children over-endow a picture with a feature of the real object a 3 (age group: 3- vs. 4- vs. 5-year-olds) ×3 (iconicity: photograph vs. colored drawing vs. schematic drawing) ANOVA was conducted with the former as between participants factor and the latter as within participant variable.

**Figure 2:**
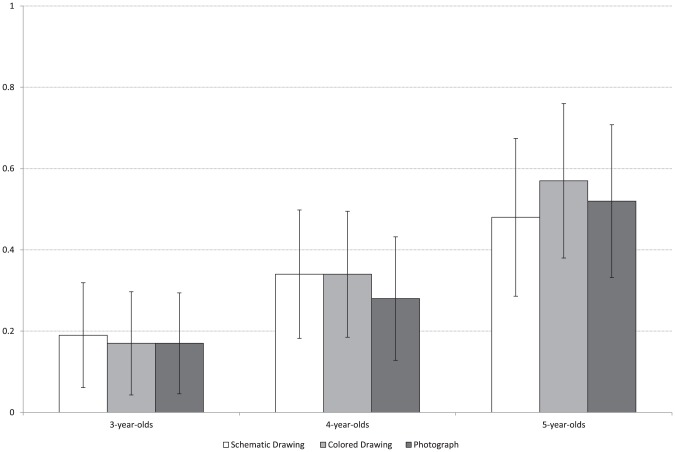
Mean performance (Range 0–1) across age groups as a function of presentation type.

Younger children were more likely to state that the picture contained the missing feature of the real object, *F*(2, 98)  = 6.96, *p* = .001, ηp^2^ = .12. Differences emerged between the adjacent ages of 3 (*M* = .17) and 4- (*M* = .32, *p* = .074, marginally significant) and in turn 5 years (*M* = .52, *p* = .051) (LSD-post hoc). Iconicity had no effect (*F*<1, *p*>.05) (Figure 2).

Thus, referential picture-object confusions reduce between 3- and 5 years when the referent changes after the picture is taken/drawn (Experiment 1) and also when the referent and the picture are different from the outset (Experiment 2). These findings indicate a general understanding of the relation between a picture state and its referent develops over preschool.

However, once a picture-object relation is established, referential confusions occur independently of iconicity of the representation. Specifically, picture-object referent confusions were equally likely to occur irrespective of whether the picture was a photograph, a colored drawing, or a schematic drawing. Power calculations of sample size [31] further indicated that there were more than sufficient participants to have an 80% chance of detecting medium sized effects. Thus, there is no evidence that iconicity plays a role in children's developing ability to treat a picture in its current physical state and what it refers to [14], [20]. Therefore, the current interpretation is that findings across both experiments may reflect representational developments [22]. A conceptual understanding of representation-referent relationship is independent of how well a picture depicts an object or a scene. Thus, picture-object domain general representational developments may underlie referential confusions.

As the first two experiments revealed that iconicity is not a factor in children's representation-referent confusions, Experiment 3 was designed to determine if children are sensitive to iconicity *per se*. That is, it is necessary to rule out the possibility that children are not affected by iconicity because they are not sensitive to it.

## Experiment 3

The aim of Experiment 3 was to investigate whether children differentiate between levels of iconicity in establishing picture-object relationships. Specifically, do children take into account the degree of resemblance when matching a representation to the correct real world referent and *vice versa*?

There is some indication that the degree of resemblance might be relevant. Three- and 4-year-old children focus on surface features when asked “what is a picture”, for example, they conceive pieces of paper with abstract form or even plain white pieces of paper as a picture. In contrast, 6-8-year-olds only regard something as a picture that represents a recognizable object [32]. This indicates a representational shift from focusing on surface features to focusing on the representational content independent of the representational medium. Thus, it is likely that children in the current experiment may focus on the level of iconicity when matching a representation to the correct real world referent.

However, we adults see pictures ‘transparently’ - generally directly through the picture-properties to the content. For example, we see a picture of a famous person in the newspaper rather than the set of coloured patches that portray the famous person. Photographs particularly enhance this transparent perception because of the high level of perceptual similarity between the referent and the photographic image [33]. Therefore, for children photographs may be treated as preferred representations for reality (*perceptual similarity hypothesis*). Conversely, photographs may make it difficult to attend to the current picture's state, in comparison to other types of pictures such as drawings. Therefore, children may be more accurate in establishing picture-object relations the more abstracted the representation (*abstracted hypothesis*). The current experiment examined how iconicity affects children's accuracy in matching a representation to its according real world referent and *vice versa*.

### Method

#### Participants

In total 83 children (31 girls) took part; 20 3-year-olds (*M* = 3.6, *SD* = 4 months), 25 4-year-olds (*M* = 4.4, *SD* = 3), 38 5-year-olds (*M* = 5.6, *SD* = 3). Children were predominately Caucasian attending local nurseries and schools with a middle class intake. All children were tested following written parental consent and their own oral assent on the day of testing. Ethical approval for this study was obtained from the Research Ethics Committee at Plymouth University.

#### Design

Each child received two conditions, i) *match picture with reality* and ii) *match reality with picture*, with a total of 6 trials. Each child was presented with 6 target objects divided into two sets (Set 1: Postman Pat and Bag, Green Mug and Spoon, and Baby Doll and Hat; Set 2: Fireman Sam and Helmet, Red Cup and Saucer, and Rag Doll and Bib). In *match picture with reality*, each iconicity type (photograph, colored drawing, and schematic drawing) was presented alongside each object. Only one acted as the correct match to the real world referent. In *match reality with picture*, each representation type was presented once alongside three referent objects (matching object with feature present, matching object but missing feature, and a distracter object). The order of conditions, the order of sets and pictorial iconicity were all counterbalanced.

#### Materials and Procedure

**Figure 3:**
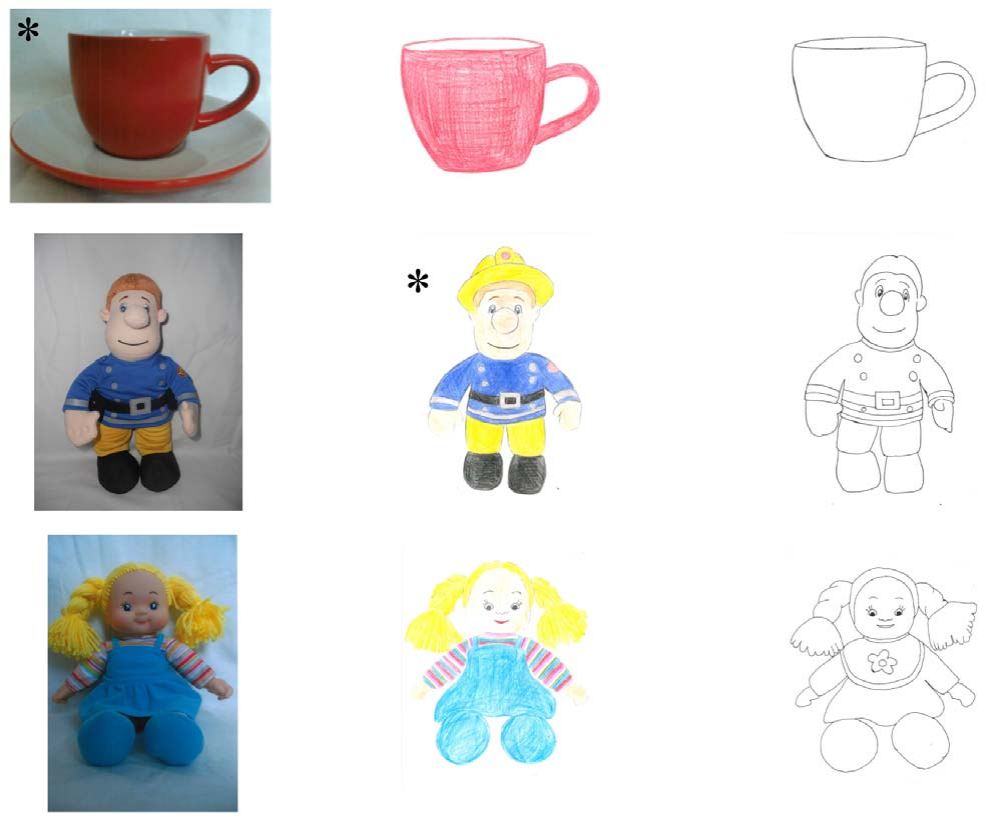
Example of Match Picture with Reality set. Only one iconicity type each matched the real-world referent (i.e., from top: photograph, colored drawing, schematic drawing).

*Match Picture with Reality.* Children were presented with an object with an additional feature (e.g., Fireman Sam with his hat) and pictures of three different iconicity types: a photo of Fireman Sam, a colored drawing of Fireman Sam and a schematic drawing of Fireman Sam. Only one of the picture representations (e.g., colored drawing) contained the additional feature (i.e., helmet) and was the correct match to the real world referent. Children were asked “Can you find the one that matches this?” *points to real world object*. This was continued with the other two objects of this set (see Figure 3 as an example).

Children scored from 0 to 3 on the number of correct matches made. Types of errors were also recorded (e.g., incorrectly selecting the photo with missing features instead of correctly selecting the schematic drawing with matching features).

*Match Reality with Picture.* Children were presented with a picture iconicity type of a real world object (e.g., colored drawing of a green mug with a spoon) in addition to three real world objects; a matching object with feature present (green mug with a spoon), a matching object with missing feature (exact same mug, no spoon) and a distracter object (different mug, no spoon) (see Table 2 for examples). Children were asked: “Find the one to match this.” *Points to picture representation*. This was continued with the other two objects of that series (i.e., Postman Pat, Baby doll).

**Table 2:** Example of Match Reality with Picture set.

Picture	Object: Matching, missing feature	Object: Mismatch, missing feature	Object: Matching, feature present
**Photograph** Postman Pat + Bag	Postman Pat - Bag	Ted Glenn - Helmet	Postman Pat + Bag
**Colored Drawing** Baby Doll + Hat	Baby Doll - Hat	Different Doll - Hat	Baby Doll + Hat
**Schematic Drawing** Green Mug + Spoon	Green Mug - Spoon	Red Mug -Spoon	Green Mug + Spoon

Children scored from 0 to 3 on the number of correct matches made. Types of errors (e.g., selecting the object with missing feature instead of the object with the feature present) were also recorded.

### Results and Discussion

To examine how children match pictures of different iconicity with real world objects and *vice versa*, two 3 (iconicity: photograph vs. colored drawing vs. schematic drawing) ×3 (age group: 3- vs. 4- vs. 5-year-olds) ANOVAs on proportional correct match scores were conducted where iconicity was the within participant variable and age group the between participants factor.

#### Match Picture with Reality

Picture-reality match was better when the photograph matched reality (*M* = .88) than both the colored drawing (*M* = .50, *p*<.001, LSD) and the schematic drawing (*M* = .38, *p*<.001, LSD) where the latter two did not differ, *F*(2, 160)  = 30.88, *p*<.001, *ηp^2^* = .28. Thus, additional color information does not play a role in children's matching of representations with their real world referents, see also Callaghan's [29] Experiment 1 for similar findings when 3-year-olds match objects to pictures. Moreover, 3-year-olds (*M* = .43) performed worse than 4-year-olds (*M* = .60, *p* = .034) who in turn performed marginally worse than 5-year-olds (*M* = .73, *p* = .057, LSD post-hoc), *F*(2, 80)  = 8.65, *p*<.001, *ηp^2^* = .18. There was no interaction (*p*>.05). That is, children across all ages performed best when the picture was perceptually most similar to the real world referent.

Importantly, 3-year-olds were above chance only when the photograph represented reality (*p*<.001), were at chance for the colored drawing (*p* = .86) and below chance for the schematic drawing (*p* = .003) (one sampled t-test: *t*(19)  = 6.35, *p*<.001; *t*(19)  = .18, *p* = .86; *t*(19)  = −3.34, *p* = .003; respectively). A similar pattern was found for 4-year-olds where photograph performance was above chance, whereas colored and schematic drawing performances were at chance (*t*(24)  = 8.29, *p*<.001; *t*(24)  = 1.09, *p* = .29; *t*(24)  = 1.47, *p* = .15; respectively). In contrast, 5-year-olds performed above chance across all iconicity levels (all at least *t*(37)>2.7, *p*<.01). Thus, iconicity affected performance in younger children more than older children.

Further, the question arises whether children have a tendency to select the photograph *per se.* If so, then this should also be reflected in the error types. Indeed, children were more likely to select the photograph (Table 3) when it was the correct representation but also compared to both when the colored drawing was correct (*χ^2^* = 21.30, *df* = 4, *p*<.001) and the schematic drawing was correct (*χ^2^* = 10.17, *df* = 4, *p* = .038). The response pattern for both colored and schematic drawing was similar (*χ^2^* = 5.63, *df* = 4, *p*>.05). Specifically, if children made an error they incorrectly selected the photograph and rarely the other alternative representation (i.e., colored vs. schematic drawing) (Table 3).

**Table 3:** Response pattern (number of children) as a function of iconicity type.

	Error: Photograph	Error: Colored Drawing	Error: Schematic Drawing	Correct
Photograph	----	5	4	74
Colored Drawing	34	----	4	45
Schematic Drawing	40	8	----	35

#### Match Reality with Picture

In contrast to above, when children matched an object to a picture, performance was very good overall, and it was irrelevant whether the picture was a photograph (*M* = .87) or a colored drawing (*M* = .81), or a schematic drawing (*M* = .83), *F*(2, 160)  = 1.01, *p*>.05, *ηp^2^* = .01. The age improvement was significant but should be interpreted with caution due to ceiling performance, *F*(2, 80)  = 4.58, *p* = .013, *ηp^2^* = .10. Three-year-olds (*M* = .73) performed worse than 5-year-olds (*M* = .90, *p* = .004, LSD post-hoc) and both did not differ from 4-year-olds (*M* = .81). Thus, matching an object to a picture is relatively easy. Our 3-year-olds' performance is comparable to a previous study in which objects were matched to pictures via a more subtle technique by placing them in a corresponding box [29]. Matching was less successful when the picture depicted an abstracted line drawing but did not differ when the picture depicted an accurate line drawing, a colored drawing, or a replica [29].

Overall, matching an object to a picture is already easy for 3-year-olds and independent of iconicity whereas the reverse is true for matching a picture to an object (i.e., reality). In particular, young children correctly focus on object features in a representation when asked to match a picture (photograph, colored drawing, schematic drawing) to a real-world referent. This finding indicates that children do not require high-iconicity pictures (perceptual similarity hypotheses) or low iconicity (abstracted hypotheses) to establish picture-object relationships. In other words, when there are no competing high-iconicity representations then low iconicity is sufficient even for 3-year-olds to accurately match pictures with objects. In contrast, young children are sensitive to iconicity that is, biased towards photographs from a choice of varying iconicity representations when asked to match a picture to a real-world referent. This was reflected in matching accuracy in both when the photograph was the correct representation, as well as error types, where children across all ages tended to select the photograph irrespective of its correctness. Particularly, the finding that young children tended not to notice when a feature of the object was missing in the photograph, suggests that the iconicity of the representation was more salient than the individual features of the object. As previously suggested, photographs are the easiest pictorial medium for children to interpret the informational meaning because they are highly perceptually similar to the real world referent [33]. Importantly, the current results suggest that children deem photographs as best representations from a choice of varying iconicity pictures for establishing an object reference *per se* even when they lack a salient feature of the referent.

## General Discussion

It is clear that children are sensitive to how well a picture represents an object in its current state. Findings from Experiment 3 demonstrate that children across all ages, given a set of pictures of differing iconicity, prefer photographs as representations for the according real world referent. Thus, the degree of resemblance does matter in construing a picture-object relationship, suggesting that children are sensitive to iconicity *per se*. This finding adds to research that has demonstrated effects of iconicity when interpretation of pictures is required. For example infants and toddlers are more likely to imitate actions depicted in highly iconic pictures (photographs) than less iconic ones (line drawings) [34] and are more likely to manually explore highly iconic pictures [35]. Importantly, the current findings show that before interpreting the meaning of pictures, iconicity is relevant in establishing a relation between a picture and what it represents in the first place.

Interestingly, once this relationship is established, that is, once children have accepted that a picture stands for a real world referent, iconicity is irrelevant as demonstrated consistently across Experiments 1 and 2. Referential confusions emerged when changes in the object were attributed to parallel changes in the picture (Experiment 1) and when they differed from the outset (Experiment 2), irrespective of whether the picture was a photograph or a colored drawing or a schematic drawing.

Could faulty source-monitoring underlie representation-referent confusion? There is a large body of evidence showing that children's discrimination of sources of events increases between 4- and 6-years [17], [18], [19]. Thus, developmentally this would fit into the current pattern of age effects. Moreover, a source memory account suggests that the higher the discriminability between events and the more varied the perceptual details, the better the memory and later source decision [16]. According to this, one would have expected better performance the lower the degree of perceptual representation-referent similarity. However, across both Experiments 1 and 2 there was no indication of more representation-referent confusion when the picture was a photograph or a colored drawing compared to a schematic drawing. Moreover, as shown in Experiment 1 memory was independent of referential confusions. Further, in Experiment 2 memory demands were kept to a minimum. That is, immediately after the picture was faced down children were asked whether the missing feature (e.g., spoon) was in the picture too. Taken together, the current findings are unlikely to be explained by source-memory developments, see also Donnelly et al. [6] for similar conclusions on referent-representation state confusions.

An alternative recent suggestion has been that children's picture-referent confusions emerge because they have difficulties in seeing photographs as static representations and that changes in the picture can “fluidly” affect the real-world referent [6]. This interpretation is based on findings that changes to the picture (e.g., pouring water over a picture) makes 3- to 4-year-olds select the object that matches the picture state. The suggestion is that low-level perceptual cues drive these errors rather than children actively believing that objects change in accordance with their representations [6]. Although this an interesting proposition, it is difficult to see how the current findings could be explained by a low-level perceptual account. If it were the case that low-level perceptual cues drove referential confusion then the current findings should have revealed different iconicity effects. It would be interesting to see whether Donnelly et al.'s [6] findings would also extend if the representation were a colored or schematic drawing.

The current findings can be better explained in terms of a representational account [22]. Previous explanations of picture-object referential confusion highlight children's difficulty in treating a picture in its current state as well as a representation of the current real world scenario [14], [20]. This by definition requires an understanding between the representational relationship between a symbol and what is refers to [9], [22]. This understanding appears to be domain general [22], [23]. Evidence for domain-generality comes from different pictorial and linguistic phenomena requiring understanding the representational relation between a stimulus and its interpretations. Specifically, around the age of 4 children develop the understanding that an ambiguous figure can have two interpretations [23], [25]. This representational pictorial understanding is related to understanding synonymy and homonymy as well as mental representations [23], [26], [27], [28]. The current findings of equal referential confusion across different representational iconicity provide direct evidence for underlying representational developments in picture-object confusion. In other words, a conceptual understanding of representation-referent relationship is independent of how well the real-world referent is depicted by the representation.

To summarize, iconicity does matter when construing the relationship between pictures of varying iconicity and an object. It does not matter in understanding the relationship between a current picture state and the real world situation it refers to. The latter is explained by general representational developments.
